# Supplementary material for: Dispersal and metapopulation stability
Source: PeerJ. 2015 Oct 1;3:e1295. doi: 10.7717/peerj.1295 (PMC4636407; doi:10.7717/peerj.1295)
Supplement: Supplemental Information 1 — Appendix 1, Continuous-time models and their analytic solutions in homogeneous metapopulations. Appendix 2, Continuous-time models with spatial heterogeneity. Appendix 3, Discrete-time models and their analytic solutions in homogeneous metapopulations. Appendix 4, Environmental stochasticity beyond white noise. [file peerj-03-1295-s001.docx]

**Dispersal and metapopulation stability**

Shaopeng Wang, Bart Haegeman and Michel Loreau

*Centre for Biodiversity Theory and Modelling, Station d’EcologieExperimentale du CNRS, 09200, Moulis, France*

Email: shaopeng.wang@ecoex-moulis.cnrs.fr, bart.haegeman@ecoex-moulis.cnrs.fr, michel.loreau@ecoex-moulis.cnrs.fr

Supplementary materials

**Appendix 1.** Continuous-time models and their analytic solutions in homogeneous metapopulations

**Appendix 2.** Continuous-time models with spatial heterogeneity

**Appendix 3.** Discrete-time models and their analytic solutions in homogeneous metapopulations

**Appendix 4.** Environmental stochasticity beyond white noise

**Appendix 5.** Supplementary figures (Figures A1-A5)

**Appendix 1.**

**Continuous-time models and their analytic solutions in homogeneous metapopulations**

In this appendix, we first explain the nature of the white-noise term in continuous-time models, and then derive the solutions for our metapopulation models in homogeneous cases.

***White-noise environmental stochasticity in continuous-time models***

In discrete time, white noise is defined as a sequence of independent Gaussian random variables. These Gaussian random variables have all zero mean and the same variance. In continuous time, the definition of white noise is more technical (van Kampen 1992). Here we do not provide a rigorous definition, but try to give an intuitive idea of continuous-time white noise. In particular, we explain how a continuous-time model with white noise (like Eqs. 1 and 2 in the main text) can be simulated numerically.

We consider a linear model,

$\frac{d\overrightarrow{X}(t)}{dt}=A\overrightarrow{X}(t)+\overrightarrow{\varpi}(t)$ (1)

with $\overrightarrow{X}(t)$ the vector of dynamical variables and *A* the matrix describing the deterministic model. The vector of white-noise perturbations $\overrightarrow{\varpi}(t)$ is characterized by a covariance matrix *V_ω_*. Because $\overrightarrow{\varpi}(t)$ is a random function, the trajectories $\overrightarrow{X}(t)$ generated by this model are also random functions. To generate a realization of $\overrightarrow{X}(t)$, we consider a (small) time step *δt* and time instants *t_k_* = *k δt* for integer *k*. We simulate the following discrete-time model,

$\overrightarrow{X}(t_{k+1})=\overrightarrow{X}(t_{k})+A\overrightarrow{X}(t_{k})\delta t+N^{*}\overrightarrow{\xi}_{k}$ (2)

The noise term $\overrightarrow{\xi}_{k}$ is a vector of Gaussian random variables with zero mean and a covariance matrix proportional to *V_ω_*. As the time step *δt* gets smaller, the discrete-time model should approximate the continuous-time model more accurately. As we show below, the limit *δt* → 0 leads to a proper continuous-time model only if the covariance matrix of the noise term $\overrightarrow{\xi}_{k}$ is proportional to *δt*. In other words, the continuous-time model we are interested in corresponds to setting the variance of $\overrightarrow{\xi}_{k}$ equal to *V_ω_δt*.

We use the discrete-time model to derive the stationary covariance matrix of $\overrightarrow{X}(t)$. In discrete-time, the covariance equation of model $\overrightarrow{X}(t+1)=A\overrightarrow{X}(t)+\overrightarrow{\varpi}(t)$ is given by *V_X_* = *AV_X_A^T^* + *V_ω_*, where *V_X_* denotes the stationary covariance matrix of vector $\overrightarrow{X}(t)$ (Van Kampen 1992). Applied to the above discrete-time model, we get (omitting one *δt*^2^ term):

$$\begin{matrix} V_{X}=(I+A\delta t)V_{X}(I+A\delta t)^{T}+V_{\varpi}\delta t \\ \begin{matrix} & = \end{matrix}V_{X}+AV_{X}\delta t+V_{X}A^{T}\delta t+V_{\varpi}\delta t \end{matrix}$$

Therefore, *V_X_* is given by the following equation:

$AV_{X}+V_{X}A^{T}+V_{\varpi}=0$ (3)

This derivation illustrates that the variance of the discrete-time noise term should be proportional to *δt*. Indeed, if the noise variance would scale as *δt^α^* with *α* > 1, then the stochastic perturbations would no longer affect the deterministic model dynamics. The solution of the covariance equation would be *V_X_* = 0, i.e., the stationary state of the model would be purely deterministic. Conversely, if the noise variance would scale as *δt^α^* with *α* < 1, then the noise would dominate. Solving the covariance equation would lead to a divergent covariance matrix *V_X_*, i.e., the deterministic model dynamics would no longer control the stochastic perturbations.

***Linearization and analytic solutions for homogeneous metapopulation models***

In all appendices, we denote the continuous-time model as *M_C_* (for comparison with discrete models), which has the form as follows:

$\frac{dN_{i}(t)}{dt}=r_{i}N_{i}(t)\cdot[1-\frac{N_{i}(t)}{k_{i}}]-d_{i}N_{i}(t)+\sum_{j\neq i} \frac{d_{j}}{m-1}\cdot N_{j}(t)+N_{i}(t)\varepsilon_{i}(t)$ (4)

In a homogeneous metapopulation without environmental fluctuations, the equilibrium local population size is simply *N*^*^ = *k*. We thus expand Eq. 4 at *N_i_*(*t*) = *N*^*^ and *ε_i_*(*t*) = 0 at first order:

$\frac{dN_{i}(t)}{dt}=-r(N_{i}(t)-N^{*})-d\cdot N_{i}(t)+\sum_{j\neq i} N_{j}(t)\cdot\frac{d}{m-1}+N^{*}\varepsilon_{i}(t)$ (5)

Denote *X_i_*(t) = *N_i_*(t) - *N^*^*, Eq. 5 can be written as:

$$\frac{dX_{i}(t)}{dt}=-(r+d)X_{i}(t)+\sum_{j\neq i} X_{j}(t)\cdot\frac{d}{m-1}+N^{*}\varepsilon_{i}(t)$$

which has matrix form:
 $\frac{d\overrightarrow{X}(t)}{dt}=J\overrightarrow{X}(t)+N^{*}\overrightarrow{\varepsilon}(t)$ (6)

where

$$J=\left(\begin{matrix} -r-d & \frac{d}{m-1} & \cdots& \frac{d}{m-1} \\ \frac{d}{m-1} & -r-d & \ddots& \vdots\\ \vdots& \ddots& \ddots& \frac{d}{m-1} \\ \frac{d}{m-1} & \cdots& \frac{d}{m-1} & -r-d \end{matrix}\right)$$

Note that *J* is the Jacobian matrix, the eigenvalues of which are: *-r* and *-r-md/(m-1)* (m-1 replicates). Local stability requires all the eigenvalues to be negative, i.e. *r* > 0. In other words, the criterion for local stability in the spatial system is the same as for the non-spatial one.

The Eq. 6 is known as an Ornstein-Uhlenbeck process (van Kampen 1992), and the covariance of $\overrightarrow{X}(t)$ (i.e. *V_N_*) is determined by the following continuous-time Lyapunov equation (see also Eq. 3):

(7)

Note that both matrices *J* and *V_ε_* have a specific form: all diagonal elements are equal and all non-diagonal elements are equal. As a result, the matrix *V_N_* also has this specific form. Therefore, to obtain *V_N_*, we only need to solve a linear equation of two unknowns. More specifically, we denote:

$J_{C}=\left(\begin{matrix} l_{1} & l_{2} & \cdots& l_{2} \\ l_{2} & l_{1} & \ddots& \vdots\\ \vdots& \ddots& \ddots& l_{2} \\ l_{2} & \cdots& l_{2} & l_{1} \end{matrix}\right)$ and

where *l*_1_ = *-r-d*, *l*_2_ = *d*/(*m*-1); *e*_1_ = *N**^2^σ^2^, *e*_2_ = *N**^2^ρσ^2^. The solution *V_N_* also has this structure:

$$V_{N}=\left(\begin{matrix} x_{1} & x_{2} & \cdots& x_{2} \\ x_{2} & x_{1} & \ddots& \vdots\\ \vdots& \ddots& \ddots& x_{2} \\ x_{2} & \cdots& x_{2} & x_{1} \end{matrix}\right)$$

From Eq. 4 we can construct two equations for the unknowns *x*_1_ and *x*_2_. We write out Eq. 4 for a diagonal element,

$$2l_{1}x_{1}+2(m-1)l_{2}x_{2}=-e_{1}$$

and for an off-diagonal element,

$$2l_{2}x_{1}+[2l_{1}+2(m-2)l_{2}]x_{2}=-e_{2}$$

These are two linear equations for *x*_1_ and *x*_2_. The solution reads,

$$x_{1}=\frac{l_{2}e_{1}-l_{1}e_{2}}{2(l_{1}-l_{2})(l_{1}+(m-1)l_{2})}$$

$$x_{2}=\frac{[2l_{1}+(m-1)l_{2}]e_{1}-2(m-1)l_{2}e_{2}}{4(l_{1}-l_{2})(l_{1}+(m-1)l_{2})}$$

By substituting the expressions for *l*_1_, *l*_2_, *e*_1_ and *e*_2_, we obtain the covariance matrix *V_N_*. Based on *V_N_*, we can calculate the variability at the multiple scales (see the main text for definitions and Table 1 for the analytic solutions).

***Dispersal-induced stability or synchrony in homogeneous metapopulatins***

When there is no dispersal (*d* = 0): (i) local alpha variability is: $\alpha_{CV}^{d=0}=\frac{\sigma^{2}}{1-(1-r)^{2}}$ in discrete models (see Appendix 3) and $\alpha_{CV}^{d=0}=\frac{\sigma^{2}}{2r}$ in the continuous model, which are equal to $\frac{\gamma_{CV}}{\phi_{e}}$ in respective models (see Appendix 3); and (ii) spatial synchrony $\phi_{p}^{d=0}=\phi_{e}$ in all models. Therefore:

$D_{\alpha}=\frac{\alpha_{CV}^{d=0}}{\alpha_{CV}}=\frac{\gamma_{CV}}{\phi_{e}}\cdot\frac{1}{\alpha_{CV}}=\frac{\alpha_{CV}\cdot\phi_{p}}{\phi_{e}}\cdot\frac{1}{\alpha_{CV}}=\frac{\phi_{p}}{\phi_{e}}$ (8)

$D_{\phi}=\frac{\phi_{p}}{\phi_{p}^{d=0}}=\frac{\phi_{p}}{\phi_{e}}$ (9)

Finally, note that *φ_p_* differ among models, therefore the dispersal-induced effects differ among the three models (see Appendix 3).

**Reference:**

van Kampen N.G. (1992). Stochastic processes in physics and chemistry. Elsevier.

**Appendix 2.**

**Continuous-time models with spatial heterogeneity**

In a two-patch metapopulation with heterogeneous local and spatial parameters, population dynamics can be described by:

$\frac{dN_{1}(t)}{dt}=r_{1}N_{1}(t)\cdot[1-\frac{N_{1}(t)}{k_{1}}]-d_{1}N_{1}(t)+d_{2}N_{j}(t)+N_{1}(t)\cdot\varepsilon_{1}(t)$ (10)

$\frac{dN_{2}(t)}{dt}=r_{2}N_{2}(t)\cdot[1-\frac{N_{2}(t)}{k_{2}}]-d_{2}N_{2}(t)+d_{1}N_{1}(t)+N_{2}(t)\cdot\varepsilon_{2}(t)$ (11)

***Stationary solution of the covariance matrix***

First, we calculate the equilibrium population size (*N*_1_*^*^, N*_2_*^*^)* by ignoring the stochastic terms (*ε*_1_*, ε*_2_). Start with randomly sampled initial population size from (0, 2) ˟ (0, 2), we simulate the population dynamics (without stochasticity) using the function *ode45* in Matlab. The processes are repeated 10 times under each set of parameters. Results show that the system always converges to same equilibrium.

Second, we calculate the Jacobian matrix around this equilibirum, which is in the following form:

$$J=\left(\begin{matrix} r_{1}(1-\frac{2N_{1}^{*}}{k_{1}})-d_{1} & d_{2} \\ d_{1} & r_{2}(1-\frac{2N_{2}^{*}}{k_{2}})-d_{2} \end{matrix}\right)$$

Finally, the stationary covariance matrix (*V_N_*) of population dynamics are given by the following Lyapunov equation (van Kampen 1992):

$JV_{N}+V_{N}J+QV_{\varepsilon}Q=0$ (12)

where *Q* is a diagonal matrix with diagonal elements of equilibrium population size, i.e. *Q* = diag{*N*_1_^*^, *N*_2_^*^}.

***A specific case with extremely asymmetric dispersal rates***

Here we examine the specific case when one population has extremely low dispersal rate, say *d*_2_ = 0. Then the dynamical equations become:

$\frac{dN_{1}(t)}{dt}=r_{1}N_{1}(t)\cdot[1-\frac{N_{1}(t)}{k_{1}}]-d_{1}N_{1}(t)+N_{1}(t)\cdot\varepsilon_{1}(t)$ (13)

$\frac{dN_{2}(t)}{dt}=r_{2}N_{2}(t)\cdot[1-\frac{N_{2}(t)}{k_{2}}]+d_{1}N_{1}(t)+N_{2}(t)\cdot\varepsilon_{2}(t)$ (14)

For population 1, it is easy to obtain its equilibrium: *N*_1_*^*^ = k*_1_(1-*d*_1_/*r*_1_). Therefore, when the dispersal rate of population 2 is zero, the dispersal rate of population 1 must be smaller than its intrinsic growth rate for local persistence. Therefore, we restrict dispersal rate to be smaller than min(*r*_1_, *r*_2_) in Figure 5. An increase in the dispersal rate of population 1 (*d*_1_) will have two consequences.

First, a higher *d*_1_ will reduce the population 1 and thereby leave the metapopulation dynamics more dominated by population 2. If the population 2 is more stable or faster (higher *r*_2_), this can increase the stability of the metapopulation (particularly when the correlation in population environmental response is low). In contrast, if the population 2 is less stable (lower *r*_2_), this will decrease metapopualtion stability.

Second, a higher *d*_1_ will increase the variability of population 1. To see this, we linearize Eq. 13 around *N*_1_ = *N*_1_^*^ and *ε*_1_ = 0:

$\frac{dX_{1}(t)}{dt}=(d_{1}-r_{1})X_{1}(t)+N_{1}^{*}\cdot\varepsilon_{1}(t)$ (15)

where *X*_1_ = *N*_1_ - *N*_1_^*^. The variance of population 1 is then easily obtained:

(16)

and consequently:

(17)

Therefore, as *d*_1_ increases (but always lower than *r*_1_), the variability of population 1 increases.

**Reference:**

van Kampen N.G. (1992). Stochastic processes in physics and chemistry. Elsevier.

**Appendix 3.**

**Discrete-time models and their analytic solutions in homogeneous metapopulations**

***Model formulas***

In discrete time, it is important to clarify the order of within-patch and between-patch dynamics (Ripa 2000). We thus develop two discrete models. The first model (*M_D_^w-b^*) is to consider that during each time step, the within-patch dynamics (described by a Ricker model) occur first, followed by the between-patch dynamics:

$N_{i}^{'}(t+1)=N_{i}(t)\cdot\exp\{r_{i}[1-\frac{N_{i}(t)}{k_{i}}]+\varepsilon_{i}(t)\}$ (18a)

$N_{i}(t+1)=N_{i}^{'}(t+1)\cdot(1-d_{i})+\sum_{j\neq i} N_{j}^{'}(t+1)\cdot\frac{d_{j}}{m-1}$ (18b)

where *r_i_* and *k_i_* represent the intrinsic growth rate and carrying capacity of patch *i*, respectively; the random variables *ε*_i_(*t*) represent environmental stochasticity in the growth rate of population *i* at time *t*. *d_i_* represents the probability for each individual in patch *i* to immigrate into other patches. In the discrete models, we assume that during each time step, the number of individuals dispersing from patch *j* into any other patch (*N_j_dj*/(*m*-1)) is smaller than those staying in patch *j* (*N_j_*(1-*d_j_*)), i.e. *d_j_* < (*m*-1)/*m*.

The second model (*M_D_^b-w^*) is to consider that during each time step, the between-patch dynamics occur first, followed by the within-patch dynamics:

$N_{i}^{'}(t+1)=N_{i}(t)\cdot(1-d_{i})+\sum_{j\neq i} N_{j}(t)\cdot\frac{d_{j}}{m-1}$ (19a)

$N_{i}(t+1)=N_{i}^{'}(t+1)\cdot\exp\{r_{i}[1-\frac{N_{i}^{'}(t+1)}{k_{i}}]+\varepsilon_{i}(t)\}$ (19b)

***Connection between discrete and continuous models***

First, it is noted that in the models *M_D_^w-b^* and *M_D_^b-w^*, the metapopulation undergoes exactly the same dynamics (i.e. ... - *growth* - *dispersal* - *growth* - *dispersal* - ...), but are censused at different times (i.e. population sizes are censused after dispersal in *M_D_^w-b^* and after local growth in *M_D_^b-w^*). Moreover, we will show these discrete-time models capture essentially the same processes with our continuous-time model (see Fig. S3-1 for an intuitive explanation).

- *Model* *M_D_^w-b^*

The discrete-time model *M_D_^w-b^* can be regarded as a specific case of the following model when *Δt* = 1:

$N_{i}^{'}(t+\Delta t)=N_{i}(t)\cdot\exp\{r_{i}[1-\frac{N_{i}(t)}{k_{i}}]\Delta t+\varepsilon_{i}(t)\sqrt{\Delta t}\}$ (20a)

$N_{i}(t+\Delta t)=N_{i}^{'}(t+\Delta t)\cdot(1-d_{i}\Delta t)+\sum_{j\neq i} N_{j}^{'}(t+\Delta t)\cdot\frac{d_{j}\Delta t}{m-1}$ (20b)

If we consider *Δt* → 0, the Eq. 20a will be (using $\exp(x)\approx1+x$ when *x* is very small):

$$N_{i}^{'}(t+\Delta t)=N_{i}(t)\cdot\{1+r_{i}[1-\frac{N_{i}(t)}{k_{i}}]\Delta t+\varepsilon_{i}(t)\sqrt{\Delta t}\}$$

Substitute into Eq. 20b, we have:

$$N_{i}(t+\Delta t)=N_{i}(t)\cdot\{1+r_{i}[1-\frac{N_{i}(t)}{k_{i}}]\Delta t+\varepsilon_{i}(t)\sqrt{\Delta t}\}\cdot(1-d_{i}\Delta t)+\sum_{j\neq i} N_{j}(t)\cdot\{1+r_{i}[1-\frac{N_{j}(t)}{k_{i}}]\Delta t+\varepsilon_{j}(t)\sqrt{\Delta t}\}\cdot\frac{d_{j}\Delta t}{m-1}$$

By ignoring higher order of *Δt* (i.e. *Δt^2^* and *Δt^3/2^*), we obtain:

$$\Delta N_{i}(t)=r_{i}N_{i}(t)\cdot[1-\frac{N_{i}(t)}{k_{i}}]\Delta t+N_{i}(t)\cdot\varepsilon_{i}(t)\sqrt{\Delta t}+[-d_{i}\cdot N_{i}(t)+\sum_{j\neq i} N_{j}(t)\cdot\frac{d_{j}}{m-1}]\Delta t$$

where $\Delta N_{i}(t)=N_{i}(t+\Delta t)-N_{i}(t)$. This is exactly our continuous-time model.

- *Model* *M_D_^b-w^*

The discrete-time model *M_D_^b-w^* can be regarded as a specific case of the following model when *Δt* = 1:

$N_{i}^{'}(t+\Delta t)=N_{i}(t)\cdot(1-d_{i}\Delta t)+\sum_{j\neq i} N_{j}(t)\cdot\frac{d_{j}\Delta t}{m-1}$ (21a)

$N_{i}(t+\Delta t)=N_{i}^{'}(t+\Delta t)\cdot\exp\{r_{i}[1-\frac{N_{i}^{'}(t+\Delta t)}{k_{i}}]\Delta t+\varepsilon_{i}(t)\sqrt{\Delta t}\}$ (21b)

If we consider *Δt* → 0, the Eq. 21b will be (using $\exp(x)\approx1+x$ when *x* is very small):

$$N_{i}(t+\Delta t)=N_{i}^{'}(t+\Delta t)\cdot\{1+r_{i}[1-\frac{N_{i}^{'}(t+\Delta t)}{k_{i}}]\Delta t+\varepsilon_{i}(t)\sqrt{\Delta t}\}$$

Substitute Eq. 21a into the above approximation, we have:

$$N_{i}(t+\Delta t)=[N_{i}(t)\cdot(1-d_{i}\Delta t)+\sum_{j\neq i} N_{j}(t)\cdot\frac{d_{j}\Delta t}{m-1}]\cdot\{1+r_{i}[1-\frac{N_{i}(t)\cdot(1-d_{i}\Delta t)+\sum_{j\neq i} N_{j}(t)\cdot\frac{d_{j}\Delta t}{m-1}}{k_{i}}]\Delta t+\varepsilon_{i}(t)\sqrt{\Delta t}\}$$

By ignoring higher order of *Δt* (i.e. *Δt^2^* and *Δt^3/2^*), we obtain again:

$$\Delta N_{i}(t)=r_{i}N_{i}(t)\cdot[1-\frac{N_{i}(t)}{k_{i}}]\Delta t+N_{i}(t)\cdot\varepsilon_{i}(t)\sqrt{\Delta t}+[-d_{i}\cdot N_{i}(t)+\sum_{j\neq i} N_{j}(t)\cdot\frac{d_{j}}{m-1}]\Delta t$$

where $\Delta N_{i}(t)=N_{i}(t+\Delta t)-N_{i}(t)$.

***Linearization and approximated solutions under homogeneous cases***

In homogeneous metapopulations where local and spatial dynamics have identical parameters (i.e. carrying capacity, intrinsic growth rates, dispersal rate, etc.), the equilibrium population size in any patch *i* is easily given by: *N** = *k*. We thus expand the models at *N_i_*(*t*) = *N*^*^ and *ε_i_*(*t*) = 0 to first order. The model *M_D_^w-b^* can be expanded as follows:

$$N_{i}(t+1)\approx[N^{*}+(1-r)(N_{i}(t)-N^{*})+N^{*}\varepsilon_{i}(t)]\cdot(1-d)+\sum_{j\neq i} [N^{*}+(1-r)(N_{j}(t)-N^{*})+N^{*}\varepsilon_{j}(t)]\cdot\frac{d}{m-1}$$

Denote *X_i_*(t) = *N_i_*(t) - *N*^*^, the above equation can be written as:

$$X_{i}(t+1)\approx[(1-r)X_{i}(t)+N^{*}\varepsilon_{i}(t)]\cdot(1-d)+\sum_{j\neq i} [(1-r)X_{j}(t)+N^{*}\varepsilon_{j}(t)]\cdot\frac{d}{m-1}$$

which has the matrix form:

$\overrightarrow{X}(t+1)\approx\Lambda_{D}[(1-r)\cdot\overrightarrow{X}(t)+N^{*}\cdot\overrightarrow{\varepsilon}(t)]$ (22)

where: $\overrightarrow{X}(t)=\left(\begin{matrix} X_{1}(t), & \cdots, & X_{m}(t) \end{matrix}\right)^{'}$

$$\vec{\varepsilon}(t)=\left(\begin{matrix} \varepsilon_{1}(t), & \cdots, & \varepsilon_{m}(t) \end{matrix}\right)^{'}$$

$$\Lambda_{D}=\left(\begin{matrix} 1-d & \frac{d}{m-1} & \cdots& \frac{d}{m-1} \\ \frac{d}{m-1} & 1-d & \ddots& \vdots\\ \vdots& \ddots& \ddots& \frac{d}{m-1} \\ \frac{d}{m-1} & \cdots& \frac{d}{m-1} & 1-d \end{matrix}\right)$$

Note that (1-*r*)*Λ_D_* is the Jacobian matrix, the eigenvalues of which are: *1-r* and *(1-r)(1-md/(m-1))* (m-1 replicates). Local stability requires all the eigenvalues to lie between -1 and 1, i.e. 0 < *r* < 2. In other words, the criterion for local stability in the spatial system is the same as for the non-spatial one.

The population dynamics reach stationary states as t → ∞. We thus calculate the covariance of both sides of Eq. 22:

(23)

where $V_{N}=Cov(\overrightarrow{X}(\infty))$ and $V_{\varepsilon}=Cov(\overrightarrow{\varepsilon}(t))$. Eq. 23 is a discrete-time Lyapunov equation. Note that both matrices *Λ_D_* and *V_ε_* have a specific form: all diagonal elements are equal and all non-diagonal elements are equal. As a result, the matrix *V_N_* also has this specific form. Therefore, to obtain *V_N_*, we only need to solve a linear equation of two unknowns (the techniques are explained in Appendix 1 for the continuous model; same techniques apply here).

Similarly, model *M_D_^b-w^* can be expanded and rewritten in the following matrix forms:

$\overrightarrow{X}(t+1)\approx(1-r)\cdot\Lambda_{D}\overrightarrow{X}(t)+N^{*}\cdot\overrightarrow{\varepsilon}(t)$ (24)

The covariance matrix *V_N_* is determined by:

(25)

which is also a discrete-time Lyapunov equation. Again, we can obtain *V_N_* by solving a linear equation of two unknowns.

With the derived covariance matrix *V_N_*, we can calculate the variability at the multiple scales (see the main text for definitions). The results are summarized in Table S3-1.

***Comparison of results under continuous-time vs. discrete-time models***

The dispersal-induced effects are much stronger when populations are censused immediately after dispersal (*M_D_^w-b^*) than after local dynamics (*M_D_^b-w^*), with those under model *M_C_* lying in between (Figs. S3-2, S3-3, S3-4). As a consequence, alpha and beta variability are always lower under model *M_D_^w-b^* than that under model *M_D_^b-w^*.

The effects of correlation of population environmental responses (*ρ*) and the number of patches (*m*) are qualitatively the same in the continuous-time and discrete-time models. However, variability changes differently with the intrinsic growth rate *r* between continuous-time and discrete-time models (Fig. S3-5). In the continuous model (*M_C_*), all measures of variability decrease monotonically with *r*. In discrete models (*M_D_^w-b^* and *M_D_^b-w^*), alpha and gamma variability and spatial variability first decrease (*r* < 1) and then increase (*r* > 1). Local regulation also weakens the synchronizing effects of dispersal and environmental correlation and thereby increases spatial asynchrony. Thus, spatial asynchrony increases with *r* in the continuous model, and first increases (*r* < 1) and then decreases (*r* > 1) with *r* in discrete models.

**Table S3-1** Analytic solutions for multi-scale variability and spatial synchrony in homogeneous metapopulations, under discrete-time (*M_D_^w-b^* and *M_D_^b-w^*) and continuous-time (*M_C_*) models. For clarity, we denote *d*' = *md*/(*m*-1) and $\phi_{e}=\frac{1+(m-1)\rho}{m}$. Note that by definition, we have *β* = *α*_cv_*/ γ*_cv_ and *φ_p_ =* 1*/β*.

|  | *M_D_^w-b^* | *M_D_^b-w^* | *M_C_* |
| --- | --- | --- | --- |
| Local-scale variability (*α_cv_*) | $\frac{\phi_{e}\cdot\sigma^{2}}{1-(1-r)^{2}}+\frac{(1-\phi_{e})\cdot\sigma^{2}}{\frac{1}{(1-d')^{2}}-(1-r)^{2}}$ | $\frac{\phi_{e}\cdot\sigma^{2}}{1-(1-r)^{2}}+\frac{(1-\phi_{e})\cdot\sigma^{2}}{1-(1-r)^{2}(1-d')^{2}}$ | $\frac{(r+d'\cdot\phi_{e})\cdot\sigma^{2}}{2r(r+d')}$ |
| Spatial asynchrony (*β*) | $1+\frac{1-(1-r)^{2}}{\frac{1}{(1-d')^{2}}-(1-r)^{2}}\cdot\frac{(1-\phi_{e})}{\phi_{e}}$ | $1+\frac{1-(1-r)^{2}}{1-(1-r)^{2}(1-d')^{2}}\cdot\frac{(1-\phi_{e})}{\phi_{e}}$ | $\frac{r/\phi_{e}+d'}{r+d'}$ |
| Metapopulation variability (*γ_cv_*) | $\frac{\phi_{e}\cdot\sigma^{2}}{1-(1-r)^{2}}$ | $\frac{\phi_{e}\cdot\sigma^{2}}{1-(1-r)^{2}}$ | $\frac{\phi_{e}\cdot\sigma^{2}}{2r}$ |
| Spatial synchrony (*φ_p_*) | 1/*β*  (see *β* above) | 1/*β*  (see *β* above) | 1/*β*  (see *β* above) |

**Figure S3-1** Comparison between the discrete-time models (*M_D_^w-b^*) and (*M_D_^b-w^*) and the continuous-time model (*M_C_*). We simulated the models until reaching the stationary state. We zoom in on a fragment of the resulting trajectories. Thick red line with circles: discrete-time model (*M_D_^w-b^*) with first local dynamics, then dispersal (time steps of this model are indicated by grey vertical lines). Thick red line with squares: discrete-time model (*M_D_^b-w^*) with first dispersal, then local dynamics. Wiggly black line: continuous-time model (*M_C_*). The discrete-time models are based on the same dynamical sequence (indicated by the thin red line), but are observed at different instants. Model (*M_D_^w-b^*) is observed after the dispersal steps (red circles), while model (*M_D_^b-w^*) is observed after the local dynamics steps (red squares). Note that these two steps have opposite effects: the local dynamics tend to drive apart the patch abundances (especially if the fluctuations applied in the patches are negatively correlated, as is the case here), while the dispersal step brings them closer together. Therefore, patch abundances are more similar in model (*M_D_^w-b^*) than in model (*M_D_^b-w^*). The continuous-time model (*M_C_*) has fluctuating trajectories whose range is comparable to the difference between the discrete-time models (*M_D_^w-b^*) and (*M_D_^b-w^*). This provides some intuition of why the three models are expected to give similar results.

**Figure S3-2** Effects of the correlation in environmental response (*ρ*) and dispersal rate (*d*) on multi-scale variability in homogeneous metapopulations, under discrete-time (*M_D_^w-b^* and *M_D_^b-w^*) and continuous-time (*M_C_*) models. Parameters: *r* = 0.5, *m* = 10, *σ*^2^ = 0.05. We restrict *d* < (*m*-1)/*m*= 0.9 so that in the discrete models, during each time step, the number of individuals dispersing from patch *i* into any other patch *j* (*N_i_d*/(*m*-1)) is smaller than those staying in patch *i* (*N_i_*(1-*d*)).

**Figure S3-3** Effects of the number of patches (*m*) and dispersal rate (*d*) on multi-scale variability in homogeneous metapopulations, under discrete-time (*M_D_^w-b^* and *M_D_^b-w^*) and continuous-time (*M_C_*) models. Parameters: *r* = 0.5, *ρ* = 0, *σ*^2^ = 0.05. For same reason as in figure A1, we restrict *d* < 0.5 (note that the lowest number of patches is 2 here).

**Figure S3-4** Dispersal-induced stability or synchrony in homogeneous metapopulations under discrete-time and continuous-time models. Black lines correspond to the continuous-time model *M_C_*, and red and blue lines correspond to the discrete-time models *M_D_^w-b^* and *M_D_^b-w^*, respectively. Note that the dispersal-induced effects are stronger when populations are censused immediately after dispersal (*M_D_^w-b^*) than after local dynamics (*M_D_^b-w^*), with those under model *M_C_* lying in between. Parameters: *ρ* = 0, *σ*^2^ = 0.05, and in (a): *r* = 0.5, *m* = 10 (solid lines) or 5 (dashed lines), in (b): *m* = 10, *r* = 0.5 (solid lines) and 1 (dashed lines) and in (c): *ρ* = 0 (solid lines) and 0.2 (dashed lines).

**Figure S3-5** Effects of the intrinsic population growth rate (*r*) on multi-scale variability in homogeneous metapopulations, under discrete-time and continuous-time models. Black lines correspond to the continuous model *M_C_*, and red and blue lines correspond to the discrete models *M_D_^w-b^* and *M_D_^b-w^*, respectively. Solid and dashed lines show results under *ρ* = 0 and 0.2, respectively. Note that when *d* = 0, the lines for the two discrete models always overlap; they also overlap with the continuous model for *β*_1_. Other parameters: *m* = 10, *σ*^2^ = 0.05.

**Appendix 4.**

**Environmental stochasticity beyond white noise**

For homogeneous metapopulations, our main result states that metapopulation variability is independent of dispersal. This result was derived for white-noise environmental stochasticity. Here we consider the extension to more general types of environmental stochasticity. In particular, we prove that the same result holds for any single-frequency perturbations (see below). This implies that the result also holds for any linear combination of single-frequency perturbations (by linearity), and thus for any noise spectrum, including white noise (in which all frequencies are equally weighted), red noise (in which low frequencies have more weight) and blue noise (in which high frequencies have more weight).

***Single-frequency perturbations***

We apply a single-frequency perturbation to the linearized system. In general, a perturbation of frequency *ω* can be written as a linear combination of two terms, one in cos(*ωt*) and another in sin(*ωt*). It is convenient to combine these two terms in a single complex-valued function proportional to e*^iωt^* = cos(*ωt*) + *i* sin(*ωt*). The linear dynamical system with a perturbation of frequency *ω* is

(26)

where $\vec{U}$ is a complex vector describing the direction in which the perturbation is applied. We use the notation $\Re(c)$to denote the real part of the complex argument *c*. Then, the stationary solution of the dynamical system is

(27)

where *I* is the unit matrix. We are interested in the dynamics of total metapopulation size *X_tot_*, equal to the sum of the components of $\vec{X}(t)$. This sum can be computed by taking the scalar product with the vector $\vec{E}=(1,1,...,1)$,

(28)

Next, we note that $\vec{E}$ is an eigenvector of matrix *J* (see main text or appendix 1 for the formula of *J*), $J\vec{E}=(-r)\vec{E}$, and also $(i\varpi I-J)^{-1}\vec{E}=(i\varpi+r)^{-1}\vec{E}$. Because *J* is symmetric, we have , and also . Hence,

(29)

Because this expression does not depend on dispersal rate *d*, we find that the dynamics of total metapopulation size *X_tot_* do not depend on dispersal rate *d*. This implies that metapopulation variability is independent of dispersal. As this holds for any perturbation frequency *ω*, it also holds for any combination of perturbation frequencies.

**Appendix 5.**

**Supplementary figures (Figures A1-A5).**

**Figure A1.** The effect of dispersal and the variance of environmental stochasticity on the gamma variability of homogeneous metacommunities, based on stochastic simulations (a, c, e) and analytic solutions from linear approximations (b, d, f). Note that (a) and (b) are of same scale, (c) and (d) are of same scale, and (e) and (f) are of same scale. Parameters: *m* = 2, *r* = 0.5, *σ*^2^ in [0.01, 0.5], *d* in [0, 1], and *ρ* = -0.9, 0 or 0.9.

**Figure A2.** Effect of spatial heterogeneities in local dynamical parameters and of (symmetric) dispersal rate on the multi-scale variability of two-patch metapopulations when environmental responses are perfectly asynchronous (*φ_e_ =* 0). The two patches differ in their intrinsic population growth rate (*r*) and/or carrying capacity (*k*), where a larger *s* indicates a higher heterogeneity. Note that the patterns of gamma variability (*γ_cv_*) have been shown in Fig. 4 (a-c).

**Figure A3.** Effect of spatial heterogeneities in local dynamical parameters and of (symmetric) dispersal rate on the multi-scale variability of two-patch metapopulations when environmental responses are perfectly synchronous (*φ_e_ =* 1). The two patches differ in their intrinsic population growth rate (*r*) and/or carrying capacity (*k*), where a larger *s* indicates a higher heterogeneity. Note that the patterns of gamma variability (*γ_cv_*) have been shown in Fig. 4 (d-f).

**Figure A4.** Effect of symmetric dispersal on the multi-scale variability in two-patch metapopulations (with homogeneous/heterogeneous local dynamics) when environmental responses are perfectly asynchronous (*φ_e_ =* 0). Note that the patterns of gamma variability (*γ_cv_*) have been shown in Fig. 5 (a-c).

**Figure A5.** Effect of asymmetric dispersal on the multi-scale variability in two-patch metapopulations (with homogeneous/heterogeneous local dynamics) when environmental responses are perfectly synchronous (*φ_e_ =* 1). Note that the patterns of gamma variability (*γ_cv_*) have been shown in Fig. 5 (d-f).
