# Supplementary figures and images for: Dispersal and metapopulation stability
Source: PeerJ. 2015 Oct 1;3:e1295. doi: 10.7717/peerj.1295 (PMC4636407; doi:10.7717/peerj.1295)

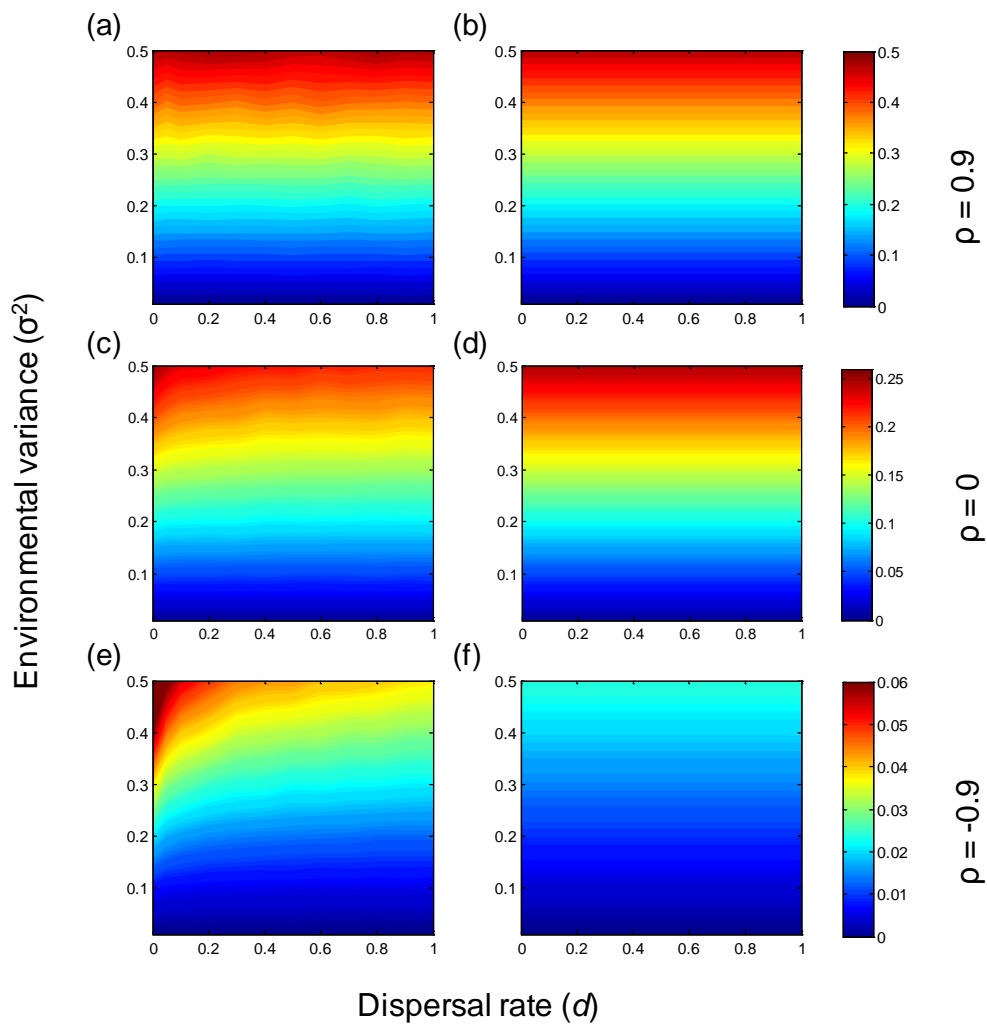

Supplement: Figure SA1 — The effect of dispersal and the variance of environmental stochasticity on the gamma variability of homogeneous metacommunities, based on stochastic simulations (A, C, E) and analytic solutions from linear approximations (B, D, F). Note that (A) and (B) are of same scale, (C) and (D) are of same scale, and (E) and (F) are of same scale. Parameters: m = 2, r = 0.5, σ2 in [0.01, 0.5], d in [0, 1], and ρ = − 0.9, 0 or 0.9. [file peerj-03-1295-s002.pdf]

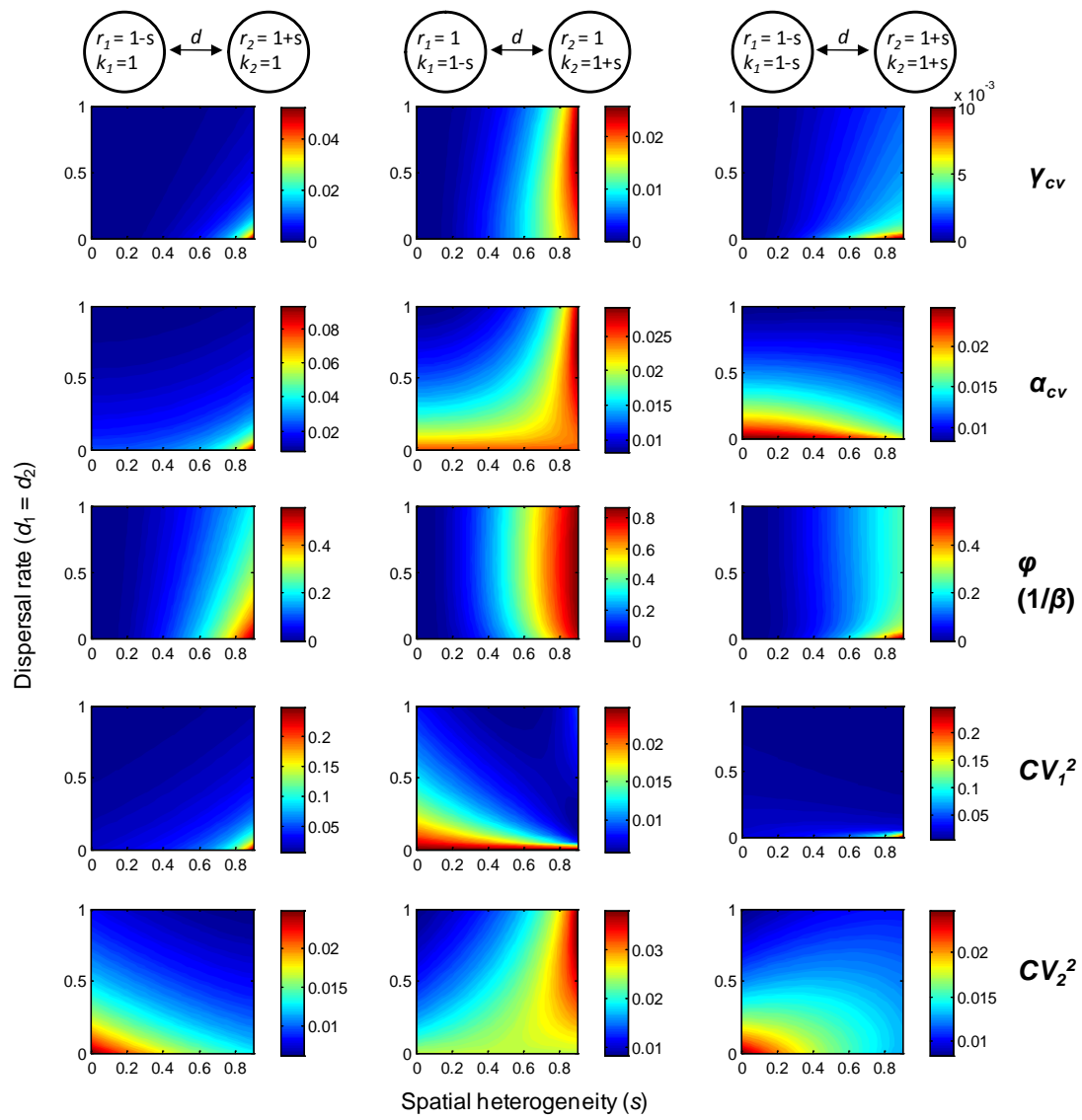

Supplement: Figure SA2 — Effect of spatial heterogeneities in local dynamical parameters and of (symmetric) dispersal rate on the multi-scale variability of two-patch metapopulations when environmental responses are perfectly asynchronous (φe = 0). The two patches differ in their intrinsic population growth rate (r) and/or carrying capacity (k), where a larger s indicates a higher heterogeneity. Note that the patterns of gamma variability (γcv) have been shown in Figs. 4A–4C. [file peerj-03-1295-s003.pdf]

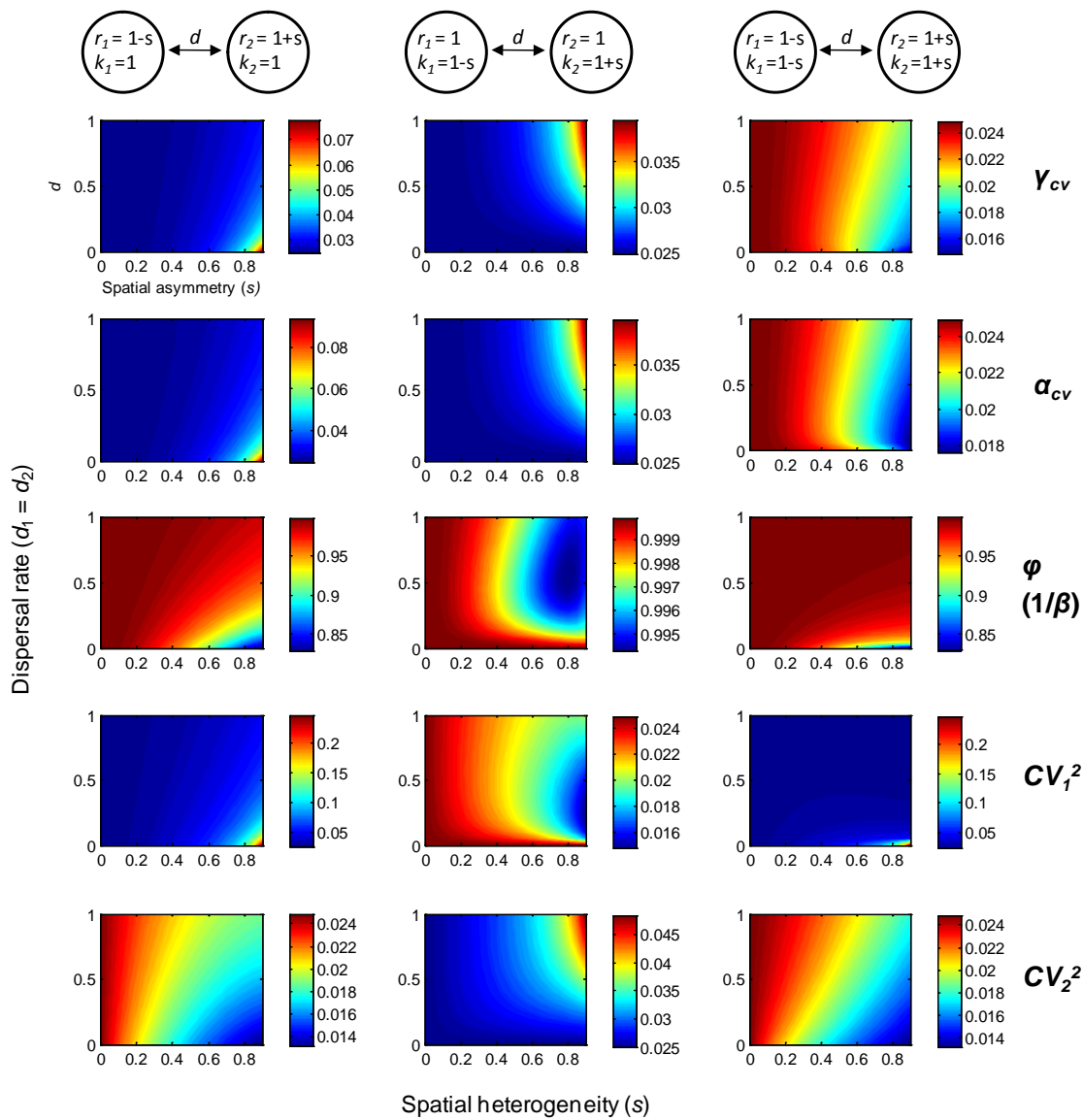

Supplement: Figure SA3 — Effect of spatial heterogeneities in local dynamical parameters and of (symmetric) dispersal rate on the multi-scale variability of two-patch metapopulations when environmental responses are perfectly synchronous (φe = 1). The two patches differ in their intrinsic population growth rate (r) and/or carrying capacity (k), where a larger s indicates a higher heterogeneity. Note that the patterns of gamma variability (γcv) have been shown in Figs. 4D–4F. [file peerj-03-1295-s004.pdf]

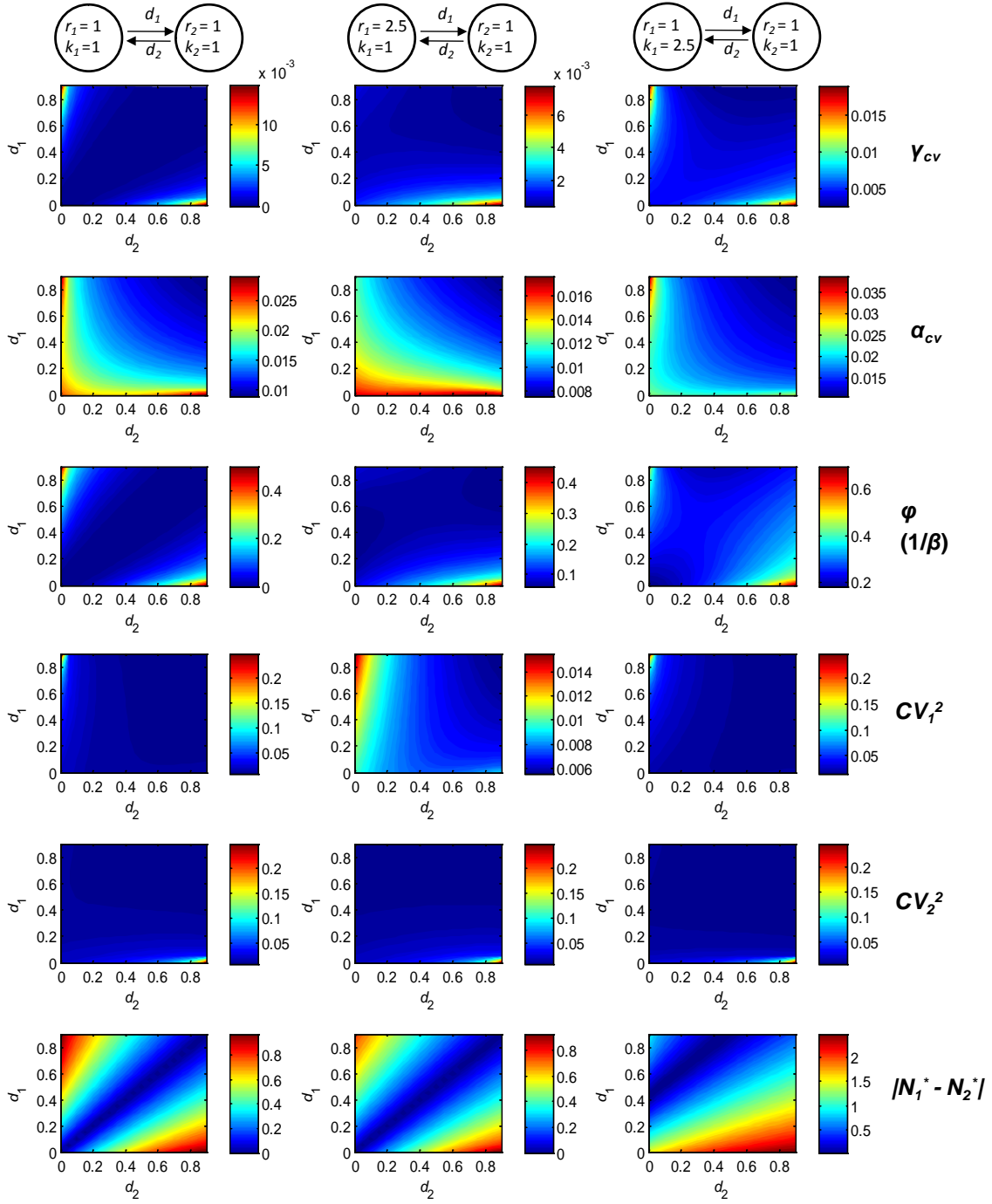

Supplement: Figure SA4 — Effect of symmetric dispersal on the multi-scale variability in two-patch metapopulations (with homogeneous/heterogeneous local dynamics) when environmental responses are perfectly asynchronous (φe = 0). Note that the patterns of gamma variability (γcv) have been shown in (Figs. 5A–5C). [file peerj-03-1295-s005.pdf]

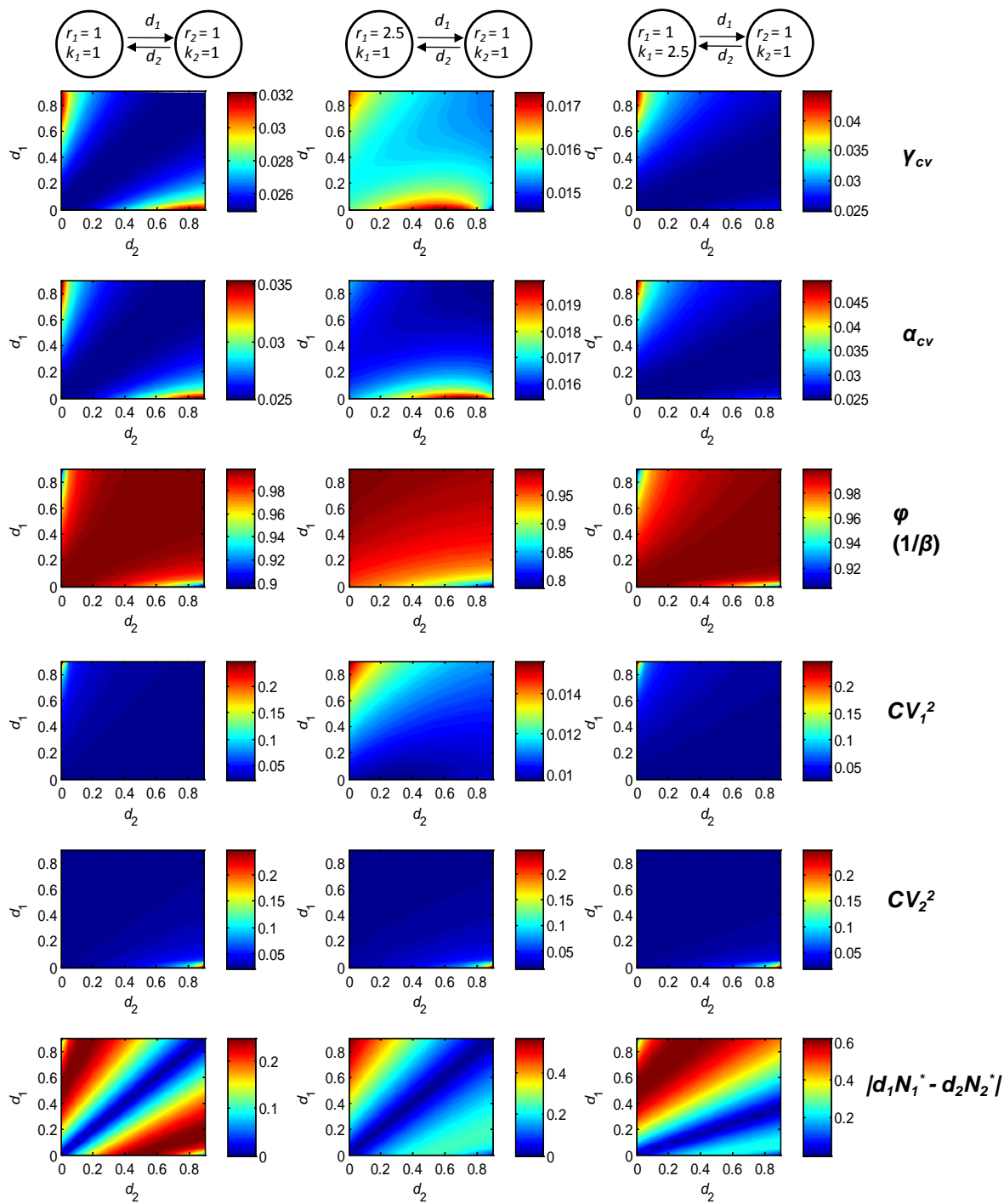

Supplement: Figure SA5 — Effect of asymmetric dispersal on the multi-scale variability in two-patch metapopulations (with homogeneous/heterogeneous local dynamics) when environmental responses are perfectly synchronous (φe = 1). Note that the patterns of gamma variability (γcv) have been shown in (Figs. 5D–5F). [file peerj-03-1295-s006.pdf]
